# Successful Isoproterenol Treatment for Ventricular Fibrillation Storm in Early Repolarization Syndrome With SCN5A Mutation

**DOI:** 10.1111/anec.70143

**Published:** 2025-12-15

**Authors:** Sung Soo Kim, Jeong Tae Byoun, Donghyeon Joo, Jum Suk Ko, Nam Ho Kim, Hyung Ki Jeong

**Affiliations:** ^1^ Department of Cardiovascular Medicine Chosun University Medical School Gwangju Korea; ^2^ Division of Cardiology, Department of Internal Medicine Wonkwang University Hospital, Wonkwang University School of Medicine Iksan Korea; ^3^ Institute of Wonkwang Medical Science Iksan Korea

**Keywords:** isoproterenol, sudden cardiac death, ventricular tachycardia

## Abstract

A 58‐year‐old man experienced a ventricular fibrillation storm with prominent inferolateral J waves and was diagnosed with early repolarization syndrome. Initial coronary angiography showed no significant stenosis and the other evaluations for ventricular fibrillation were unremarkable. Despite conventional therapy for ventricular fibrillation, it recurred. Isoproterenol infusion suppressed the J wave and successfully mitigated ventricular fibrillation episodes. This case highlights the role of isoproterenol in managing early repolarization syndrome‐related ventricular fibrillation storms and the possible pathogenic link between SCN5A mutations and J wave syndromes.

## Graphical abstract

Suppression of VF storm in ERS with SCN5A mutation using isoproterenol.

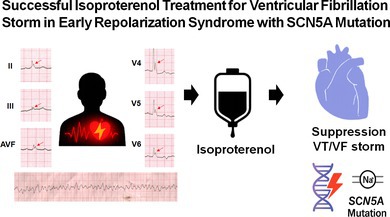

## 1 Case Report

**FIGURE 1:**
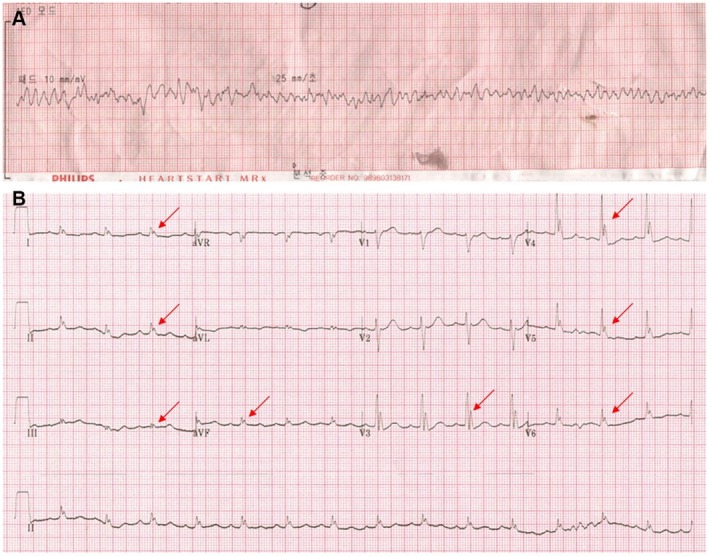
(A) Automated external defibrillator electrocardiogram (ECG) showing ventricular fibrillation. (B) ECG obtained at coronary care unit showing a prominent J wave in inferolateral leads (red arrow).

**FIGURE 2:**
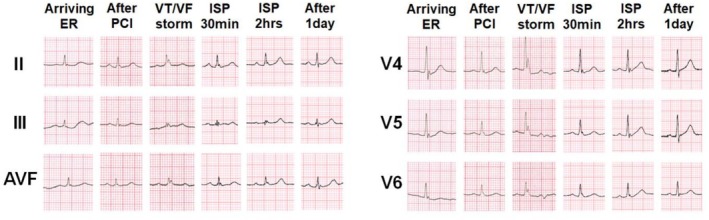
Serial changes in electrocardiogram. The J wave in inferior leads (II, III, AVF) and lateral leads (V4, V5, V6) change over time and in response to isoproterenol infusion. ER, emergency room; ISP, isoproterenol; PCI, percutaneous coronary intervention; VF, ventricular fibrillation; VT, ventricular tachycardia.

A 58‐year‐old man presented to the emergency room (ER) with cardiac arrest. He experienced several episodes of loss of consciousness in his twenties and thirties. However, he had not undergone any medical evaluation at that time. The patient had no family history of sudden cardiac death (SCD). The patient had been taking medications for hypertension and diabetes mellitus. At 3 a.m., the patient experienced abnormal breathing with gasping sounds while sleeping. The patient's spouse attempted to wake him up; however, he did not respond. The spouse called emergency medical system and initiated chest compression. An electrocardiogram (ECG) from an automated external defibrillator (Figure 1A) showed ventricular fibrillation (VF), prompting the device to deliver an electronic shock. VF recurred three more times, necessitating additional defibrillations. On examination, blood pressure was 100/50 mmHg, pulse rate was 80/min, respiratory rate was 22/min, and body temperature was 36.1°C. Troponin I was 13 ng/mL (0–14 ng/mL), CK‐MB was 1.4 ng/mL (0–5.1 ng/mL), CK was 62 IU/L (32–187 IU/L), and BNP was < 10 pg/mL (0–150 pg/mL). Furthermore, creatinine was 1.0 mg/dL (0.6–1.2 mg/dL), Na was 137 mEq/L (135–150 mg/dL), and K (3.5–5.5 mg/dL) was 3.9 mEq/L. Chest radiography revealed nonspecific findings with a normal cardiac shadow. The ECG in the ER did not reveal any significant changes, such as ST elevation. Coronary angiography revealed focal stenosis of approximately 70% of the distal right coronary artery. However, the distal coronary flow was preserved. A coronary stent was placed at the lesion site. The patient was then transferred to the coronary care unit for intensive monitoring. During monitoring, the J wave became more prominent, particularly in the inferolateral leads (Figure 1B). After 3 h, the VF recurred. Despite defibrillation, VF continued to recur. The ECG after conversion to sinus rhythm showed no ischemic changes, such as ST elevation, T inversion, or Q waves; however, the J wave remained prominent. Owing to the VF storm, an endotracheal tube was inserted for mechanical ventilation, and the patient was deeply sedated. Propranolol was administered. However, VF continued to occur repeatedly. Because the ECG showed no evidence of myocardial ischemia but displayed prominent J waves in the inferolateral leads, early repolarization syndrome (ERS) was suspected. Consequently, isoproterenol was infused to mitigate the VF storm. Following isoproterenol administration, the J wave disappeared (Figure 2), and VF did not recur. The patient was further evaluated to determine the underlying cause of the VF storm. Myocardial single‐photon emission computed tomography (SPECT) and signal‐averaged ECG revealed no pathological changes. Cardiac magnetic resonance image (MRI) confirmed the absence of myocardial ischemia. Additionally, genetic testing using next‐generation sequencing (NGS) for arrhythmias demonstrated an SCN5A mutation, suggesting that VF was likely caused by ERS.

## 2 Discussion

Here, we present a case of a VF storm successfully alleviated with isoproterenol in a patient with coronary artery lesions with ERS and an SCN5A mutation. Despite appropriate reperfusion therapy and sedation, VF storm recurred. After the infusion of isoproterenol, the J wave disappeared and VF did not recur.

In the current guidelines, general management of ventricular arrhythmia storm suggests identifying the cause and withdrawal, correction of reversible cause, sedation, and beta‐blockers for relieving sympathetic tone. In addition to the general management, specific inherited arrhythmic syndromes require tailored therapeutic approaches.

ERS is characterized by a J point elevation with a prominent J wave at the terminal portion of the QRS complex and has traditionally been regarded as benign (Mehta and Jain 1995). However, experimental and clinical studies have suggested a natural fatal course (Gussak and Antzelevitch 2000; Haïssaguerre et al. 2008; Nam et al. 2008).

J waves are generally known to be associated with hypothermia. However, these can also be observed in conditions such as myocardial ischemia, electrolyte imbalance, and Brugada syndrome. The present case showed normal body temperature and potassium levels. The exact mechanism of J waves and the subsequent development of fatal arrhythmias is not fully understood. Theories suggest either depolarization or repolarization defects. However, this topic is beyond the scope of this study. Briefly, VF in ERS has been associated with gene mutations that affect the cardiac action potential. These include gain‐of‐function mutations in the ATP‐sensitive potassium channel (KCNJ8) and loss‐of‐function mutations in the L‐type calcium (CACNA1C, CACNB2, and CACNA2D1) and sodium channel (SCN5A) activity, as observed in our patient (Barajas‐Martínez et al. 2012; Burashnikov et al. 2010; Koncz et al. 2014). These gene mutations typically shift the net electronic ion current outward. Therefore, a transmural voltage gradient results in electrical heterogeneity and induces fatal ventricular arrhythmia.

Medical treatments that might counteract inward currents, such as isoproterenol, which is known to enhance the calcium inward current, and quinidine, which is known to inhibit transient outward potassium currents, have been reported to be treatments and are recommended according to the current guidelines (Zeppenfeld et al. 2022).

Coronary artery disease (CAD) is the most common cause of SCDs (Myerburg and Junttila 2012). In the present case, a coronary angiogram was performed to rule out CAD. A focal lesion in the distal right coronary artery was identified and percutaneous coronary intervention was performed because of the significance of the lesion. Whether the coronary lesion caused the VF recurrence remains uncertain. This may have also encouraged or triggered electrical instability. However, considering that the distal flow was not significantly compromised, recurrent VF might be attributed to another pathogenesis. Notably, further evaluations, including myocardial SPECT, cardiac MRI, and signal‐averaged ECG, suggested that ischemia was unlikely to be the direct cause of the VF storm. Given the prominent J waves in the inferolateral leads on the ECG, the cause of VF was likely to be ERS. This was further supported by genetic test results, which revealed an SCN5A mutation (c.1579G>A; p.Gly527Arg, heterozygous). Because there are no large‐scale data available in Korea regarding the association between SCN5A mutations and ERS, the NGS results were classified as variants of uncertain significance (VUS).

Additional context regarding this variant may help interpret its clinical relevance. The c.1579G>A (p.Gly527Arg) missense variant in SCN5A replaces a neutral glycine with a positively charged arginine at codon 527. While this substitution could alter the structure or function of the Nav1.5 sodium channel, in silico predictions such as PolyPhen‐2 and REVEL suggest possible pathogenicity—placing the variant in an indeterminate range (REVEL score 0.5–0.7)—these results remain inconclusive (Ioannidis et al. 2016; Wehrens et al. 2003). This variant is currently classified as a VUS in ClinVar and is extremely rare in the general population (8 out of 243,942 alleles in gnomAD). It has also been reported in a small number of clinical cases, including individuals with Brugada syndrome and epilepsy (Campuzano et al. 2020; Li et al. 2020; Pablo Flórez et al. 2018). Although its clinical significance remains uncertain and additional evidence, including functional and population‐level studies, will be necessary to clarify any potential role in ERS or VF susceptibility, the variant's physicochemical properties and location raise the possibility of a functional effect.

In the present case, isoproterenol administration successfully treated a VF storm due to ERS, especially in a patient with a SCN5A gene mutation, which was suspected to be the cause of ERS. In cases of refractory VF with ERS, despite appropriate management, isoproterenol can be a favorable treatment option for patients with J wave syndrome.

## Author Contributions

Sung Soo Kim, Jeong Tae Byoun contributed writing‐original draft, conceptualization, data curation, approval of the final version of the manuscript, and agreement of all aspects of the work. Hyung Ki Jeong and Donghyeon Joo performed the analysis with discussion. All authors read and approved the final manuscript.

## Ethics Statement

Written informed consent was obtained from the patient and this study was approved by the Wonkwang University Hospital Institutional Review Board (2024‐11‐003).

## Conflicts of Interest

The authors declare no conflicts of interest.

## Data Availability

The data that support the findings of this study are available on request from the corresponding author. The data are not publicly available due to privacy or ethical restrictions.
